# Perceptions of and hesitancy toward COVID-19 vaccination in older Chinese adults in Hong Kong: a qualitative study

**DOI:** 10.1186/s12877-022-03000-y

**Published:** 2022-04-06

**Authors:** Judy Yuen-man Siu, Yuan Cao, David H.K. Shum

**Affiliations:** 1 Department of Applied Social Sciences, Faculty of Health and Social Sciences, The Hong Kong Polytechnic University, Hong Kong, People’s Republic of China; 2 Interdisciplinary Centre for Qualitative Research and Training, The Hong Kong Polytechnic University, Hong Kong, People’s Republic of China; 3 Research Centre for SHARP Vision, The Hong Kong Polytechnic University, Hong Kong, People’s Republic of China; 4 Department of Rehabilitation Sciences, Faculty of Health and Social Sciences, The Hong Kong Polytechnic University, Hong Kong, People’s Republic of China; 5 Research Institute for Smart Ageing, The Hong Kong Polytechnic University, Hong Kong, People’s Republic of China; 6 Research Institute for Intelligent Wearable Systems, The Hong Kong Polytechnic University, Hong Kong, People’s Republic of China

**Keywords:** Perceptions, Barriers, Incentives, Vaccines, COVID-19, Critical medical anthropology, Chinese, Hong Kong

## Abstract

**Background:**

COVID-19 vaccination is recommended for older adults by the World Health Organization. However, by July 15, 2021, only 26% of individuals over 60 years old in Hong Kong had received a first dose of the vaccine. The health belief model and the theory of planned behavior have been used to understand the determinants for COVID-19 vaccination in past literature. However, vaccination determinants can be complex and involve social and cultural factors that cannot be explained by micro-individual factors alone; hence, the health belief model and the theory of planned behavior cannot provide a complete understanding of vaccine hesitancy. Few studies on the barriers to, hesitancy toward, and motivations for COVID-19 vaccination among older Chinese adults have been performed. The aim of this study is to fill this gap by conducting a comprehensive analysis of this subject using the critical medical anthropology framework, extending the health belief model and the theory of planned behavior in understanding vaccination determinants among the older adult population.

**Methods:**

Between November 2020 and February 2021, 31 adults (24 women and 7 men) over the age of 65 took part in semi-structured, one-on-one interviews. The data we gathered were then analyzed through a phenomenological approach.

**Results:**

Two major themes in the data were examined: barriers to vaccination and motivations for vaccination. The participants’ perceptions of and hesitancy toward vaccination demonstrated a confluence of factors at the individual (trust, confidence, and social support networks), microsocial (stigma toward health care workers), intermediate-social (government), and macrosocial (cultural stereotypes, civic and collective responsibility, and economic considerations) levels according to the critical medical anthropology framework.

**Conclusions:**

The decision to receive a COVID-19 vaccination is a complex consideration for older adults of low socioeconomic status in Hong Kong. Using the critical medical anthropology framework, the decision-making experience is a reflection of the interaction of factors at different layers of social levels. The findings of this study extend the health belief model and the theory of planned behavior regarding the understanding of vaccination perceptions and relevant behaviors in an older adult population.

## Background

According to the World Health Organization (WHO), as of September 10, 2021, 223,022,538 cases of COVID-19 and 4,602,882 COVID-19-related deaths have been confirmed worldwide; and the numbers are still increasing [1]. In an effort to end the pandemic, health authorities around the world have encouraged people to receive vaccinations against COVID-19 [2, 3]. As of September 2021, 13 vaccines have been approved by the WHO [3]. In Hong Kong, citizens can choose between the Pfizer-BioNTech and Sinovac-CoronaVac vaccines, which are mRNA and inactivated vaccines, respectively [4]. Older adults in Hong Kong have been urged to receive COVID-19 vaccination because they are at higher risk of both requiring hospitalization and intensive care and of dying if infected [5]. However, the majority of older adults in Hong Kong are vaccine-hesitant, which has led to delays in COVID-19 vaccination [6]. By July 15, 2021, only 26% of adults in Hong Kong aged 60 years or older had received a first dose of a COVID-19 vaccine [7].

### Significance

The health belief model (HBM) and theory of planned behavior (TPB) have been widely used to understand the determinants of COVID-19 vaccination [8–12]. However, vaccine hesitancy is a complex, multifaceted construct rooted in the sociocultural structures that guide decision-making [13]. Therefore, an examination of the elements of the HBM (perceived susceptibility, perceived severity, perceived benefits, perceived barriers, cues to action, and self-efficacy) and the TPB (attitude, subjective norms, behavioral intention, perceived behavioral control, and perceived power) [14] that is mostly focused on personal and individual attributes is inadequate when working to understand vaccination behaviors. The determinants of vaccination involve lived experiences, given that people’s experiences can affect how they behave and perceive the world around them. This study was therefore conducted using a phenomenological approach that aligns with the critical medical anthropology (CMA) framework. The CMA framework has been used to explain older adults’ behaviors concerning vaccination against seasonal influenza and pneumococcal diseases [15]. Under the CMA framework, a person’s health-related behaviors and perceptions are influenced by factors at the individual, microsocial, intermediate-social, and macrosocial levels [16]. At the individual level, personal factors and social support networks influence a person’s health-related behaviors and perceptions [15]. At the microsocial level, the interaction between a person and their health care provider influences the person’s health-related behavior [16]. Policy, ethnocultural and religious beliefs, and socioeconomic status within a capitalistic ideology jointly affect a person’s health-related behaviors and perceptions at the intermediate-social and macrosocial levels [16].

Furthermore, most studies on vaccine motivations and barriers have been quantitative [9, 10, 17]. The few qualitative studies concerning barriers to COVID-19 vaccination have focused on young adults [18–21], and they cannot be generalized to older adults. The perceptions of older Chinese adults toward COVID-19 vaccination and the hesitancy of older people to receive the vaccination are therefore poorly understood. This issue poses a pressing concern, because older adults are particularly vulnerable to COVID-19 infection and its effects [5]. The underlying reasons for this population’s hesitancy to receive the vaccination must be understood to enable health authorities to design a vaccination promotion strategy targeting older adults.

## Methods

A qualitative research approach was adopted in this study. During the study period, individual, semi-structured, in-depth interviews were conducted with 31 Hong Kong residents (24 women and 7 men) aged 65 years or older. Data saturation was achieved, and no new themes and codes emerged, which confirms the validity of the themes and the conclusions that emerged in the study [22].

### Ethical considerations

Ethics approval was obtained from the Human Subjects Ethics Subcommittee of the Hong Kong Polytechnic University before the study began (ID: HSEARS20200827001-04). All the procedures were performed in accordance with relevant guidelines and regulations. The participants were informed of the study procedures before they were interviewed, and written informed consent was obtained from all of them.

### Data collection

Thirty-one older adults were recruited using purposive sampling with the following criteria: they (1) were Hong Kong citizens, (2) were aged 65 years or older, (3) had not received the COVID-19 vaccine by the time of the study, and (4) were able to communicate in Cantonese Chinese at a satisfactory level of fluency and comprehension. The criteria were formulated to ensure that the participants had had a long period of exposure to Hong Kong society and to minimize the potential effects of other cultural factors. At the time of the study, older adults comprised the largest population group in Hong Kong that remained unvaccinated against COVID-19, because only 1.5% of those aged 80 years or older, 5.4% of those aged 70 to 79 years, and 14.4% of those aged 60 to 69 years had received a first dose [23].

Of the 31 participants, 30 were recruited from two nongovernmental organizations (NGOs), namely the Hong Kong Lutheran Social Service (LC-HKS) and the Hong Kong Society for Rehabilitation (HKSR), the two largest NGOs offering services to older adults in Hong Kong. Recruiting participants from these NGOs ensured that the sample was representative of the elderly population in Hong Kong. Managers at the LC-HKS assisted in recruiting participants from centers for older adults located in five districts, namely, Shatin, Tsuen Wan, Tuen Mun, San Po Kong, and Chai Wan, which are distributed across the most densely populated areas in Hong Kong (Kowloon Peninsula, Hong Kong Island, and the New Territories). The HKSR, which provides old-age and rehabilitation services to more than 1.3 million people each year, distributed information on this study to its members. Interested individuals registered by completing an online form. The research assistant screened potential participants using the sampling criteria and invited them to participate in an interview.

To ensure that the interviews were administered consistently, the participants were interviewed individually by the same research assistant between November 2020 and February 2021. The research assistant did not know the participants personally before, which helped ensure that the interviews were conducted with minimal bias. The research assistant has a background in applied psychology at both the undergraduate and master’s levels and has extensive experience in administering interviews through a certificate in counseling. To ensure the quality of the interviews, the research assistant received intensive training from the first author. All of the authors supervised the research assistant throughout the data collection process and provided guidance as necessary. To facilitate interaction, all the interviews were conducted in Cantonese Chinese, the native language of both the interviewer and the participants.

An interview question guide was used to direct the focus of the interview discussions. As developed with reference to the literature on perceptions of and barriers to vaccination [18–21], the guide used an inductive design and did not contain assumptions about the data collection procedure. The questions addressed macrolevel and microlevel factors that may have influenced the participants’ decision-making experiences regarding vaccination. To offer the participants flexibility in expressing their views and experiences, the questions were open-ended [24].

Because the interviews were conducted during the ongoing COVID-19 pandemic, the participants could opt to be interviewed online instead of in person. To protect participant confidentiality, the face-to-face interviews were held either in a private room at the authors’ institution or at one of the LC-HKS’s centers for older adults. Six interviews were conducted face to face, and 25 interviews were conducted online. Each interview lasted 1–1.5 h and was audio recorded with the participant’s consent. As compensation for their time, each participant was given a supermarket cash coupon worth HKD200 (approximately USD 26) upon completion of the interview.

### Data analysis

The interviews were transcribed verbatim, and a phenomenological approach was used to investigate the meaning and essence of the participants’ lived experiences [25]. The first author read through all of the transcripts thoroughly to ensure familiarity with the content and then conducted a second readthrough to identify the major themes [26]. Next, the transcripts were analyzed line by line using an inductive coding process that segmented the transcripts into small meaning units, which were then labeled and categorized [26, 27]. Upper-level categories were determined based on the research questions, and in vivo coding was conducted, to highlight recurrent categories [26]. Through repeated examination and comparison, overlapping codes and categories were consolidated into themes encompassing similar concepts [26]. The codes, categories, and themes derived from the data, along with supporting interview quotes, were then organized into a coding table [28].

### Study rigor and data reliability

Data collection and analysis were conducted in compliance with the guidelines for the Consolidated Criteria for Reporting Qualitative Research [29]. The criteria developed by Lincoln and Guba were used to ensure the rigor of the study design and methods [30]. Data saturation was achieved, because the redundancy in the data indicated that no new themes or codes would emerge from further interviews [22, 27]. Quotes from the interviews were included in the coding table to ensure that the codes were grounded in the interview data, and cross-checking between the interview quotations, themes, and categories was performed throughout the analysis. The coding procedure was conducted by the first author, and a consensus on the coding was reached among all three authors.

## Results

### Participant characteristics

The demographics of the participants are in Table 1. All of the participants were ethnically Chinese. Of the 31 participants, 19 were willing to receive COVID-19 vaccinations, 6 refused, and 6 were hesitant. Of the 19 participants willing to receive a vaccination, 5 had previously received a vaccination against seasonal influenza, and of the 12 participants who either refused (*n* = 6) or were hesitant (*n* = 6) to receive a COVID-19 vaccination, 6 had previously received a vaccination against seasonal influenza. This distribution of participants was conducive to the investigation of both the incentives for and barriers to vaccination.

**Table 1:** Demographics of the participants

Informant Code	Sex	Age	Education level	Work status	Job nature	Marital status	Monthly income (in $HK)	Social security assistance receiving	Family caregiver	Housing type	District of residence	Willingness to receive COVID-19 vaccination Y = Yes N = No H = Hesitant
E02	M	65–74	Below primary	Retired	NA	Married	< 4000	Yes (not specified)	Yes	Rental Public Housing	Kwai Tsing	H
E04	F	75–79	Below primary	Housewife	NA	Married	0	Comprehensive Social Security Assistance	No	Rental Public Housing	Shatin	N
E05	F	75–79	Below primary	Housewife	NA	Divorced	0	Comprehensive Social Security Assistance	Yes	Rental Public Housing	Shatin	Y
E07	M	70–74	Junior secondary	Retired	NA	Married	0	Comprehensive Social Security Assistance	No	Subdivided flats	Tsuen Wan	Y
E11	F	70–74	Junior secondary	Housewife	NA	Single	0	Comprehensive Social Security Assistance	No	Rental Public Housing	Shatin	Y
E12	F	80–84	Junior secondary	Housewife	NA	Married	0	Comprehensive Social Security Assistance	No	Rental Public Housing	Shatin	Y
E15	F	70–74	Junior secondary	Housewife	NA	Widowed	0	Old Age Allowance	No	Self-owned Home Ownership Scheme Housing	Island East	N
E16	F	70–74	Junior secondary	Housewife	NA	Widowed	0	Old Age Allowance	No	Rental Home Ownership Scheme Housing	Island East	N
E18	M	65–69	Junior secondary	Retired	NA	Divorced	0	Old Age Allowance	No	Rental Public Housing	Kwun Tong	Y
E19	F	80–84	Junior secondary	Retired	NA	Widowed	0	Old Age Allowance	No	Rental Public Housing	Island East	Y
E20	M	> 85	Senior secondary	Retired	NA	Married	0	Old Age Allowance	No	Self-owned Home Ownership Scheme Housing	Island East	Y
E21	F	75–79	Senior secondary	Retired	NA	Married	0	Old Age Allowance	No	Self-owned Home Ownership Scheme Housing	Island East	N
E22	F	75–79	Junior secondary	Retired	NA	Widowed	0	Old Age Allowance	No	Self-owned Home Ownership Scheme Housing	Island East	Y
E24	M	75–79	Junior secondary	Retired	NA	Married	0	Old Age Allowance	No	Rental Public Housing	Tuen Mun	Y
E26	F	80–84	Senior secondary	Retired	NA	Widowed	0	Old Age Allowance	No	Self-owned Private Housing	Tuen Mun	Y
E27	F	65–69	Junior secondary	Retired	NA	Widowed	0	Comprehensive Social Security Assistance	No	Rental Public Housing	Tuen Mun	H
E28	F	65–69	Refuse to answer	Housewife	NA	Widowed	0	Comprehensive Social Security Assistance	No	Self-owned Public Housing	Tuen Mun	N
E29	F	65–69	Below primary	Part time job	Domestic helper	Widowed	< 4000	Comprehensive Social Security Assistance	No	Self-owned Home Ownership Scheme Housing	Tuen Mun	H
E31	F	65–69	Below primary	Retired	NA	Married	0	Old Age Allowance	No	Rental Public Housing	Tuen Mun	H
E32	F	70–74	Below primary	Retired	NA	Married	0	Old Age Allowance	No	Rental Public Housing	Tuen Mun	H
E34	F	65–69	Junior secondary	Retired	NA	Married	0	Comprehensive Social Security Assistance	No	Rental Public Housing	Sai Kung	Y
E35	F	65–69	Junior secondary	Retired	NA	Widowed	0	Comprehensive Social Security Assistance	No	Self-owned Home Ownership Scheme Housing	Wong Tai Sin	Y
E36	M	75–79	Senior secondary	Retired	NA	Married	0	Comprehensive Social Security Assistance	No	Rental Public Housing	Sai Kung	Y
E38	F	70–74	Senior secondary	Retired	NA	Widowed	0	Old Age Allowance	No	Self-owned Home Ownership Scheme Housing	Wong Tai Sin	Y
E39	F	65–69	Junior secondary	Housewife	NA	Married	0	Old Age Allowance	No	Self-owned Home Ownership Scheme Housing	Wong Tai Sin	Y
E40	F	75–79	Junior secondary	Retired	NA	Married	0	Old Age Allowance	No	Self-owned Home Ownership Scheme Housing	Wong Tai Sin	Y
E44	F	> 85	Below primary	Retired	NA	Refuse to answer	0	Comprehensive Social Security Assistance	No	Rental Public Housing	Wong Tai Sin	Y
E45	F	> 85	Below primary	Retired	NA	Widowed	0	Old Age Allowance	No	Rental Public Housing	Kwun Tong	H
E46	F	65–69	Below primary	Retired	NA	Single	0	Old Age Allowance	No	Rental Public Housing	Wong Tai Sin	N
E47	F	65–69	Junior secondary	Retired	NA	Single	0	Comprehensive Social Security Assistance	No	Rental Public Housing	Kowloon City	Y
E48	M	80–84	Below primary	Retired	NA	Married	0	Old Age Allowance	No	Self-owned Home Ownership Scheme Housing	Wong Tai Sin	Y

The participants represent older Hong Kong adults of low socioeconomic status. All 30 of the participants who opted to answer questions regarding their socioeconomic status received social welfare assistance from the government through either Comprehensive Social Security Assistance (*n* = 13) or an Old Age Allowance (*n* = 17); the remaining participant refused to indicate the type of social welfare assistance that he was receiving. Most of the participants were retired (*n* = 22) or were homemakers (*n* = 8); only two participants received some form of income. Most of the participants were living in government-subsidized housing: rental public housing (*n* = 17), self-owned housing under the Home Ownership Scheme (*n* = 10), rental housing under the Home Ownership Scheme (*n* = 1), and self-owned public housing (*n* = 1). One participant was living in a subdivided flat (a flat that is shared by several households). Only one participant was living in self-owned private housing. Ten participants had not finished primary school, 15 had received junior secondary school education, and 5 had received senior secondary school education.

### Barriers to and hesitancy toward COVID-19 vaccination

The 12 participants who refused (*n* = 6) or were hesitant (*n* = 6) to receive COVID-19 vaccination frequently mentioned that they experienced barriers to and feelings of hesitancy toward vaccination. Barriers to and hesitancy toward vaccination can be explained by factors at the individual, microsocial, intermediate-social, and macrosocial levels under the CMA framework.

#### Factors at the individual level

##### Lack of trust and confidence in the vaccine

A lack of trust and confidence in the vaccine was a common barrier to vaccination. Although some of the participants had previously been vaccinated against seasonal influenza, they held different perceptions of the COVID-19 vaccine. Several factors affected the participants’ confidence in the vaccine, with its short development time commonly cited as a concern:

The period of research on the pneumonia [COVID-19] vaccine was too short. I usually get the flu vaccine, and have participated in a study on it over the past 3 years, but that new flu vaccine still isn’t yet ready. How can the pneumonia [COVID-19] vaccine come out so quickly? It’s too fast. I’m doubtful and not confident about it. [E21]

The participants also viewed the COVID-19 vaccine as overly new, given that relatively few people had received it. This led to hesitancy toward vaccination:

The vaccine was developed too quickly, and I don’t think that many people have got vaccinated. You can’t possibly know what will happen to you after you get vaccinated. I want to wait for more people to get vaccinated and see how they respond. If everyone who gets vaccinated is fine, then I may get vaccinated as well. [E16]

##### Perceptions of the vaccine being dangerous

Almost all of the vaccine-hesitant and refusing participants were worried about the safety of the vaccine, with side effects mentioned as the most common concern:

I would not consider getting vaccinated at this point. I am not sure what would happen to me if I got vaccinated. I read in the newspapers that someone ended up with a twisted face [Bell’s palsy] after getting vaccinated. It’s really frightening and scary. Newspapers report that many people experienced side effects after getting vaccinated overseas. For now, I won’t get vaccinated; I’ll let others do it first. If they’re fine, I might reconsider. [E21]

Perceptions of vaccine side effects were not always drawn from reports or the media, and some were based on the participants’ past experiences with vaccination:

I am afraid of the vaccine because I get a fever every time when I receive a vaccination. I end up being sick for at least 3 days. The [pneumococcal] vaccine that I got several years ago made me sick. I think this [COVID-19] vaccine would make me sick too. [E21]

Fear induced by reports of death after COVID-19 vaccination was another barrier:

I don’t want to get vaccinated because I don’t know what will happen to me; not everyone is suited to receiving the vaccine. Someone died after getting vaccinated. Will that happen to me too? I don’t know. I heard that 3 out of 100 people die after getting vaccinated. Who knows whether I’ll be a lucky one? I don’t want to die yet. [E04]

##### Perceptions of poor long-term effectiveness

Most vaccine-hesitant and refusing participants believed that the vaccine was only effective for a short period, which reduced their motivation to receive a vaccination:

The vaccine is only effective for 3 months. Why get vaccinated? What happens after 3 months? The immunity [you get] from the vaccine is not for life. There is no point to get a vaccine with such a short effectiveness. [E28]

##### Perceptions of being unsuitable for vaccination

Although some of the participants had been vaccinated against seasonal influenza, many of them perceived themselves to be unsuitable candidates for COVID-19 vaccination. This perception was partly due to existing chronic conditions:

I have been vaccinated against the flu and against pneumonia [pneumococcus]. However, I’m still deciding whether to get this [COVID-19] vaccine because many problems after getting vaccinated have been reported overseas. I have chronic conditions […], so I think I would have a higher chance of [experiencing] side effects. It wouldn’t be a problem if I can go [die] quickly after vaccination; but if it [dying] takes a long time, or if I cannot go [die] but suffer from serious after-effects, it would be agonizing. [E31]

Some of the participants thought that they were unsuitable for vaccination due to their age:

I am still deciding [whether to get vaccinated], and I want to see how others respond first. I’m old, so I don’t know what will happen to me if I get vaccinated. Many people say that they experience discomfort or serious side effects after vaccination. Therefore, I want to wait for more old people to try the vaccine first to see the impact. As I am old, I really have no idea about what impacts the vaccine would have on me, so I have to be cautious. [E45]

##### Peer pressure

The participants’ social networks were a source of peer pressure, affecting their motivation to get vaccinated:

Many of my friends who are around my age have said that they want to wait and observe [the situation] a while longer. My friends’ opinions affect my confidence in the vaccine, making me wonder if the vaccine is actually good. The vaccine isn’t a must for me, so I would also prefer to wait a while […]. It’s not only my friends; some other older people who I have met in my volunteer work have said that they would rather wait and see how others respond to the vaccine first. Since most people at my age think in this way, I also want to wait and observe [the situation] longer, as I have no strong thoughts about getting vaccinated. [E29]

##### Fragile social networks

Some of the participants had fragile social networks due to their age and lack of social connections. These participants were concerned that no one would help them if they experienced side effects from the vaccine. This constituted a strong barrier to vaccination:

I won’t get the vaccine because I don’t know what will happen to me. Not everyone is suitable for the vaccine, and some people have died from it. How can I know whether I’m suitable [for the vaccine]? If I have side effects from the vaccine, who will take care of me? If I die at home, who will be able to help? I would be very worried if I got vaccinated. [E46]

Fragile family networks were another barrier. The participants indicated that they were concerned about burdening their family members if they experienced serious side effects:

I’m chronically ill, so I’m more cautious because I’m afraid of [experiencing] side effects from vaccination. Even worse, if the side effects were really severe and lasting for a long time, but I didn’t die, I would burden my family members. They’re young and have to work. Who would have time to take care of me? […] If you’re sick for a long time, you’ll be abandoned. [E31]

#### Factors at the microsocial level

##### Stigma toward health care workers

In general, the participants perceived contact with health care workers to be risky during the ongoing pandemic. This perception constituted a formidable barrier to vaccination:

I avoid seeing doctors and nurses as they are dangerous because they are working in hospitals. They may have come in contact with the pneumonia [COVID-19]. When I attend follow-up appointments at the hospital, I feel afraid, but I have no choice because I need the medication. Vaccination is done by doctors and nurses, but it is optional, so I will try to avoid it as much as possible. [E27]

#### Factors at the intermediate-social level

##### Lack of trust in the government

Lack of trust in the government also weakened the participants’ motivation to receive vaccination:

I do not know whether the vaccine works or not. If it’s really necessary, I think civil servants should get it first. Then, I might consider it. However, I doubt that the vaccine for civil servants is the real vaccine. Civil servants can pretend to get vaccinated, but they might not be getting the real vaccine. The real vaccine must be harmful, so I don’t think civil servants would take that risk. The vaccine for us ordinary citizens would be the real, harmful one. [E28]

To increase public trust in the vaccine, the Hong Kong government established the Expert Committee on Clinical Events Assessment Following COVID-19 Immunization to provide an independent assessment of potential causal links between adverse events and COVID-19 vaccines. If adverse events are found to be linked to the COVID-19 vaccine, monetary compensation is given to those affected. However, even the Expert Committee could not alleviate the concerns of some of the participants. Their lack of confidence in the vaccine is due to the belief that the government and the Expert Committee will refuse responsibility (and thus refuse to award monetary compensation) for side effects experienced after vaccination:

There are many cases of side effects caused by the vaccine. However, the government and the experts [Expert Committee] always say that the side effects aren’t caused by the vaccine. They say that the side effects are due to those people’s chronic conditions. If you have side effects, they just say it’s your own problem. It seems like the government doesn’t want to take responsibility and give [these people] compensation. I think it would be better for me not to get vaccinated. [E16]

#### Factors at the macrosocial level

##### Perceptions of vaccines as toxic

Cultural influences caused half of the vaccine hesitant and refusing participants to believe that the vaccines were toxic, decreasing their motivation to receive vaccination:

Although I know that I’m in a high-risk group, I would rather not get vaccinated because I might experience side effects. I heard that the vaccine may lead to “withered bones” [avascular necrosis]. Vaccines, just like medication, have side effects because, after all, they are toxic. Therefore, I’m afraid to get vaccinated. It is a fact that the vaccine is toxic to the bones, and my bones are bad already. If I get vaccinated, then my bones would get even worse. [E15]

##### Perceptions of vaccination as a viral injection

Some of these participants believed that being vaccinated involved the injection of a virus into their body, a thought that was unacceptable to them:

I don’t feel comfortable with vaccination. The vaccine is a virus. When you get vaccinated, you are actually injecting the virus into your body. I really can’t accept the idea of injecting something bad into the body. [E28]

### Motivations for receiving COVID-19 vaccination

Nineteen of the participants indicated that they were considering vaccination. Their motivation to receive the COVID-19 vaccination can also be explained under the CMA framework by factors at the four social levels.

#### Factors at the individual level

##### Trust in the vaccine

A high level of trust in the vaccine was a common motivator for the participants who were considering vaccination:

I have no special preference for any brand [of the COVID-19 vaccine]. As long as there is a vaccine for me, that’s good enough. I trust that the vaccines aren’t developed with bad intentions. They’re doing good for people and aren’t hurting people. Also, when vaccines are made available to the public, I trust that they have already passed all the tests and experiments and have been proven to be safe for public use. [E20]

Confidence in the vaccine was instrumental in motivating the participants to receive vaccinations. One of the participants expressed confidence in the vaccine because the chief executive of Hong Kong had received it:

Yes, I will definitely get vaccinated. I’ve already made an appointment and will get vaccinated soon. The chief executive has got vaccinated already. She’s not afraid to get vaccinated, so why should I hesitate? She is old, and I’m old too. We’re similar in age, and she had no problems after getting vaccinated. That means the vaccine is safe for old people. [E47]

The participants also expressed high levels of confidence in the vaccine due to awareness of global vaccination levels:

Yes, I’ll get vaccinated. I have registered already. I want to get vaccinated to obtain peace of mind. I do not want to get infected and trouble others. Besides, I am confident in the vaccine because so many people around the world have got vaccinated already, and most of them are fine. The high government officials have all got vaccinated too, so what do I have to be worried about? The vaccine was created for people’s benefit. It targets the virus; it was not created to harm people. I understand the vaccine is not 100% safe, but if so many people around the world have got vaccinated already, why shouldn’t I? [E48]

##### Obtaining a sense of security

The participants reported that a strong motivator for receiving COVID-19 vaccination is the sense of security they feel it will bring:

I think that the vaccine can prevent the pneumonia [COVID-19]. Getting vaccinated will give me peace of mind. I’m living with my family, so getting vaccinated will help me feel safer living with them. You know, the disease can be transmitted easily among people living together. Also, I won’t need to worry or feel anxious every time I go out. Getting vaccinated is just like a heart-stabilizing pill [Cantonese slang meaning something that brings peace of mind]. [E40]

The development of the vaccine was a source of hope for some of the participants:

I will get vaccinated because the vaccine is our only hope. I hope that the vaccine can really work for us. I hope that the vaccine can really be effective. I hope that the vaccine can really kill the virus—they said the antibodies from the vaccine can kill the virus. [E47]

##### Influence of family members

Positive perceptions of vaccination among the participants’ family members increased the participants’ motivation to receive vaccination:

I will see what my younger family members think about the vaccine first. I don’t have a strong opinion on it. If they think it’s fine to get vaccinated, then I’ll go do it. Many of my friends think the same way. [E29]

Intentions to visit family members overseas also motivated some of the participants:

Getting vaccinated can give me peace of mind. Also, I hope that I can leave Hong Kong to visit my relatives overseas. You know, if you want to go overseas, you have to get vaccinated first. [E48]

The perceptions of the participants’ peers were also influential:

I’ll see what my friends do first. If many of my friends get vaccinated, and if many other people get vaccinated, then it is easier for me to ask for opinions, and I’ll follow their choice and get vaccinated. If very few of my friends get vaccinated, I would have few friends to ask for opinion about which vaccine I should choose. [E26]

#### Factors at the microsocial level

##### Trust in medical experts

The opinions of medical experts affected the participants’ motivation for being vaccinated:

I will ask my doctor’s opinion before getting vaccinated because I trust him. Even the government needs to consult medical experts. These experts have done a lot of research on the vaccine and think that the vaccine is good, so the government is encouraging us to get vaccinated. If the experts said the vaccine is good and reliable, why not trust them? That’s why they’re the experts; you have to listen to them. I don’t think the experts would deceive the government. The experts who made the vaccines are helping people, not harming people. [E40]

#### Factors at the intermediate-social level

##### Trust in the government

The participants with higher levels of trust in the political regime were more motivated than those with lower levels of trust in receiving the vaccination. The belief that “the government won’t harm people” was frequently expressed:

I will definitely get vaccinated. I’ll just do what the government asks me to. All governments should have good intentions and do good for their citizens. I don’t think any government would intentionally harm their people. Therefore, you should trust your government. The government is very good. It has paid so much money to buy vaccines for us. What more can you expect? [E05]

#### Factors at the macrosocial level

##### Vaccination as a civic responsibility

Most of the participants who perceived receiving vaccination as a social responsibility were willing to be vaccinated:

I will get vaccinated. If you want the society to go back to normal, everyone should take the responsibility and get vaccinated. You shouldn’t think that you don’t need to get vaccinated just because others will receive the vaccine. If everyone thinks that way, then we’ll stay stuck like we are now. [E39]

The belief that receiving vaccination was a civic responsibility was closely related to the participants’ desire for a return to pre-pandemic normalcy:

If there were no pneumonia [COVID-19] and the society were like how it was before, that’d be very good. At least people would be able to get a job, especially people working in tourism. Right now, people working in tourism are really in bad shape—they lost their jobs because of the epidemic […]. If the vaccine is out and if everyone gets vaccinated, then we can restart the economy, and our society will be able to go back to normal. [E07]

##### Getting infected as a collective responsibility

To control the spread of the pandemic, the Hong Kong government issued quarantine orders. In addition to those who are infected, those who have had contact with a confirmed case are also required to undergo quarantine and compulsory COVID-19 virus testing for a specific time period. This policy caused some of the participants to perceive that they would trouble others if they became infected. Avoiding getting infected, thus, was perceived as a collective responsibility. This belief strongly motivated vaccination:

I’ll get vaccinated if I can, because then I won’t infect others. It wouldn’t be a big deal for me to get infected, but I’d feel guilty if I infected others or caused others to have to quarantine. A friend of mine just told me that someone with pneumonia [COVID-19] was in a restaurant that she had been to, so she and her husband had to stay at Penny’s Bay [Quarantine Centre] for 2 weeks. That is really bad for making others involved. [E44]

##### Free vaccination

The fact that the COVID-19 vaccine is free served as a motivator for vaccination:

I think that the vaccine is effective. People spending that much time and effort on developing a vaccine should be trying to help people, right? Frankly, the vaccine is very expensive, and our government could choose not to provide the vaccine to us for free, right? The vaccine is free now, so why not get vaccinated? We are able to have the vaccine for free because the upper [government] cares much about the citizens of Hong Kong. [E22]

## Discussion

Two major themes emerged from the participants’ experiences regarding COVID-19 vaccination, namely, barriers to vaccination and motivations for vaccination, which can be analyzed through factors at the four social levels considered under the CMA framework.

### Individual level

At the individual level, the participants’ personal experiences and social networks strongly affected both their barriers to and incentives for vaccination. For those participants open to vaccination (*n* = 19), their trust in the vaccine, their desire to achieve a sense of security, and positive experiences with the vaccine among members of their social networks were significant motives. This group of participants tended to have a strong belief that COVID-19 vaccines have been developed for the good of the people. Those participants who refused (*n* = 6) or were hesitant (*n* = 6) to be vaccinated, by contrast, exhibited more distrust in the safety and efficacy and expressed a negative view of the vaccine due to weak social and family networks. The hesitant participants frequently noted that they would delay their decision to be vaccinated until others had received the vaccine. This result is consistent with findings from previous research [31]. Some of the hesitant participants wished to delay their COVID-19 vaccination because they wanted to spend time deciding on the “best” vaccine, that is, the vaccine with the highest efficacy and the fewest side effects.

Vaccine hesitancy has been widely documented in the literature [32, 33]. Vaccine acceptance should be interpreted on a continuum spanning from complete acceptance of all vaccines to complete refusal of all vaccines, with hesitancy in between [7]. The 3C model, consisting of complacency (which arises from perceptions that the risks from diseases preventable through vaccination are low), convenience (concerning the physical availability and financial affordability of the vaccine), and confidence (regarding trust in the safety and effectiveness of the vaccine), has been proposed as a method to understand vaccine hesitancy [7]. This model was later modified and extended to the 5C model for the psychological antecedents of vaccination: confidence, complacency, constraints (availability, affordability, and accessibility), calculation (engagement in seeking information), and collective responsibility (willingness to protect others) [34]. However, because the causes and mechanisms underlying the elements of the models remain unknown, both the 3C and 5C models are inadequate for developing a robust understanding of vaccine hesitancy. Based on the results of the analysis, it can be asserted that social, cultural, economic, and political factors influence peoples’ perceptions of and behavior regarding vaccination.

Studies have presented conflicting results on the influence of prior exposure to the seasonal influenza vaccine with respect to their motivation to receive the COVID-19 vaccine. Wang et al. [35] reported that having previously received vaccinations against seasonal influenza enhanced motivation to receive the COVID-19 vaccine in China, whereas Malik et al. [36] observed no association in their study in the United States. The results of this study do not reveal a clear association between the two types of vaccination. Of the 19 participants who were willing to receive a COVID-19 vaccination, only 5 had previously received seasonal influenza vaccinations. Of the 12 participants who refused (*n* = 6) or were hesitant (*n* = 6) to receive COVID-19 vaccination, 6 had received vaccination against seasonal influenza. A prior experience of receiving a seasonal influenza vaccine did not guarantee a greater acceptance of COVID-19 vaccination among the study participants. Although both vaccines are provided free of charge to older adults in Hong Kong (seasonal influenza vaccine is provided with government subsidies to those who are aged 50 or above under the Vaccination Subsidy Scheme), the vaccination rates for both vaccines remain low. The vaccination rates for seasonal influenza among people aged 65 or older made up 45.8%, 44.7%, and 39.3% of that population in 2019/2020, 2020/2021, and 2021/2022, respectively [37]. Older adults have the lowest rate of COVID-19 vaccination in Hong Kong; in July 2021, only 26% of adults aged 60 years or older had received their first dose [6]. Although the COVID-19 vaccination rate among older adults has increased dramatically in 2022 (85.25% for those 60–69 years old, 74.16% for those 70–79 years old, and 45.07% for those 80 years old or older have been vaccinated with the first shot as of late February 2022), the vaccination rate is still among the lowest at the time of this article when compared to younger age groups (excluding the 3 to 11 age group, which only received approval for vaccination in February 2022) [23]. Some of the participants indicated that the free vaccination was an incentive for receiving vaccination, but the results suggest that financial factors are not the sole motivator for obtaining a vaccination among older adults.

Trust in the vaccine has been identified as a major factor in individuals’ decisions regarding vaccination [38], and trust and confidence in the COVID-19 vaccine were identified as key determinants of motivations for and barriers to vaccination for the participants in this study. The participants’ confidence in the COVID-19 vaccine was undermined by its novel nature and short development time. The participants who refused or were hesitant toward the vaccine perceived that the short development timeline of the vaccine indicated potential harmful effects, such as adverse vaccination events. “I can’t possibly know what will happen to me [if I get vaccinated]” was a common concern among the participants. The hesitant participants stated that they would delay COVID-19 vaccination to observe its effects in others. Similar perceptions are prevalent in the U.S., where vaccine skepticism remains a major obstacle to achieving herd immunity [39]. Among the participants with high acceptance of COVID-19 vaccination, trust in the vaccine, in medical experts, and in the government was commonly expressed. These participants typically believed that the COVID-19 vaccine was developed to help rather than to harm the public.

Consistent with the findings of studies on motivations for vaccination [40, 41], social support networks comprised of peers and family members could provide both incentives for and barriers to vaccination. Vaccine acceptance by peers and family members in particular contributed to strong motivation for receiving vaccination. The participants who refused or were hesitant toward vaccination had peers and family members who were hesitant about receiving the vaccine. Furthermore, the participants with fragile family networks were often hesitant to receive vaccination because they were concerned that they would not be able to obtain assistance and would be left alone if they experienced side effects or sequelae. The fear of burdening family members in the event of experiencing severe side effects or chronic sequelae was also a notable barrier to vaccination. Overall, the participants’ social support networks were critical in both increasing and decreasing their levels of vaccine acceptance. In 2018, 15.7% of individuals aged 65 years or older in Hong Kong lived alone [42], and the fragile family and social networks of this group of older adults may contribute to their low levels of vaccination. Furthermore, a recent report noted that the “hidden elderly,” or those without family and social support, are vulnerable to isolation during the pandemic, making obtaining support regarding vaccination difficult [43]. The participants in this study were from lower socioeconomic classes, which are shown to be closely associated with poor social support networks [44]. Governmental health authorities and public health practitioners must consider social support as a key factor when promoting COVID-19 vaccination in older adults. Strengthening social support networks for older adults or providing additional support after vaccination may reduce this population’s hesitancy toward vaccination and increase their motivation to receive the vaccine.

### Microsocial level

At the microsocial level, stigma regarding health care workers and perceptions that these individuals were dangerous due to potential contact with the COVID-19 virus were notable demotivating factors. The participants who refused or were hesitant to receive COVID-19 vaccinations perceived contact with health care workers during the ongoing COVID-19 pandemic to be risky. Specifically, health care workers were stigmatized by these participants as “dirty” (in cultural and non-physical terms) because they work in hospital environments, which the participants perceived as contaminating. If people are considered “dirty” or “unclean,” they may be ostracized by others [45]. The stigma attached to health care workers therefore served as a barrier to vaccination for those opposed to or hesitant toward receiving the vaccine.

### Intermediate-social level

At the intermediate-social level, political factors related to trust in the government influenced the participants’ decisions to receive vaccinations. Acceptance of the vaccine was higher among the participants with high levels of trust in the government, and a lack of trust in the government was noted as a demotivating factor by those opposed to or were hesitant toward the vaccine. Purity, liberty, and antiauthority are values associated with vaccine hesitancy, with liberty and antiauthority specifically relating to a lack of trust in the government [46]. Past studies note that the political affiliation of the past presidents of the United States can have a significant impact on its citizens’ vaccine acceptance or hesitancy. One study noted that endorsement of the COVID-19 vaccine by then-U.S. President Donald Trump did little to encourage vaccine acceptance among the U.S. public [47]. Another national survey conducted in the U.S. observed that public confidence in and acceptance of the vaccine increased after one of former President Barack Obama’s daughters received the vaccination [48]. This study also reveals the contribution of political factors to vaccination behavior. The participants who expressed lower levels of trust in the government also had doubts about the authenticity of the vaccine. Furthermore, although the Hong Kong government has established an expert committee to investigate adverse events following COVID-19 vaccination [49], some of the participants continued to doubt the safety and effectiveness of the vaccine. They believed that the adverse events experienced by some individuals after receiving the vaccine had been dismissed as being explainable by those individuals’ pre-existing chronic conditions. These participants believed that neither the government nor medical experts were held accountable for occurrences of adverse effects related to the vaccine and that neither were willing to award monetary compensation for these effects. This idea undermined the participants’ confidence in the vaccine, contributing to their negative perceptions of it. In sum, the attribution of adverse events arising after COVID-19 vaccination to the pre-existing chronic conditions of those affected was perceived by these participants as an excuse and a refusal of responsibility for harm done by the government.

### Macrosocial level

At the macrosocial level, the participants’ cultural perceptions of vaccines help explain their hesitancy to receive vaccination. The results align with the finding presented in a systematic review by Wilson et al. [50], who found that cultural factors may serve as barriers to vaccination. The participants who refused or were hesitant toward vaccination viewed vaccines as “toxic” (i.e., dangerous). This perception is common in Chinese societies [16, 51] and was reinforced by these participants’ past experiences of feeling ill after vaccination.

Studies have reported that a sense of responsibility to society has been a key motivator of individuals’ infection control behavior during the ongoing COVID-19 pandemic [9, 34, 52]. Awareness of the social consequences of COVID-19 can encourage people to adopt prosocial and altruistic behaviors, such as receiving vaccination [53]. Moral obligations can also strengthen justifications for vaccination policies [54], thereby further promoting vaccination. This leaning is reflected in the findings, given that a sense of civic responsibility and unwillingness to cause trouble for others were two strong incentives for vaccination in participants willing to receive the vaccine. The participants believed that they had a collective and civic responsibility to not only avoid infection but also to take action (i.e., receive vaccination) to prevent infection and illness and enable society to return to normal. Per Hong Kong’s infection control policy (as of February 2022), people who come into close contact with COVID-19 patients (e.g., family members living in the same household, colleagues working in the same office, or dining partners) must quarantine at the Penny’s Bay Quarantine Centre. Individuals living in the same building as individuals with COVID-19 must also be quarantined, albeit less strictly, and undergo compulsory viral testing. This feeling of collective responsibility therefore serves as an effective measure for containing infection and as a motivator for vaccination. Furthermore, some of the participants perceived vaccination as a means to protect themselves from the moral blame that they could experience if they contracted COVID-19 and caused those around them to undergo quarantine procedures.

The COVID-19 vaccination rate among older adults increased dramatically in 2022 when the fifth wave of COVID-19 hit Hong Kong; 85.25% of those 60–69 years old, 74.16% of those 70–79 years old, and 45.07% of those 80 years old and older have been vaccinated with the first shot as of late February 2022 [23]. This increase in vaccination is due in part to the Hong Kong government’s recent implementation of policies to address the barriers to and enhance the motivators for vaccination at various social levels. For example, support networks for older adults living in residential care premises have been introduced by health care providers (individual-level management). These health care providers provide proactive physical evaluations for these older adults living in residential care premises, which helps increase their trust in health care workers (microsocial level management). Measures to encourage the general public to get vaccinated can also promote a positive atmosphere around vaccination, serving to exert peer pressure on older adults (individual level management). Furthermore, the introduction of the Vaccine Pass on February 24, 2022 [55] enhances peer pressure regarding vaccination (individual level management) and reinforces the idea that receiving COVID-19 vaccination is a civic responsibility (macrosocial level management). These measures to tackle the barriers to and enhance the motivators for vaccination at different social levels of the CMA framework may serve to increase the acceptance of vaccination in the older adult population.

### Limitations

These findings should be interpreted with caution, because they are based on interviews with a small sample of older adults (31 participants) who were recruited from two NGOs located in five districts of Hong Kong. Although data saturation was achieved, the findings of this study have limited generalizability and thus cannot represent the older adult populations of other communities. Confidence in the results can be strengthened through follow-up studies involving a greater number of participants recruited from more sites.

Given that the data for this study were collected between November 2020 and February 2021, the rising vaccination rate among older adults seen in 2022, when the fifth wave COVID-19 hit Hong Kong, is not reflected.

## Conclusions

The results reveal that the decision to receive COVID-19 vaccination is a complex consideration for older adults of low socioeconomic status in Hong Kong. Under the critical medical anthropology framework, the decision-making experience is a reflection of the interaction of factors at the individual (trust, confidence, and social support networks), microsocial (stigmatization of health care workers), intermediate-social (government), and macrosocial (cultural stereotypes, civic and collective responsibility, and economic considerations) levels. The findings of this study extend the health belief model and the theory of planned behavior regarding the understanding of vaccination perceptions and relevant behaviors in an older adult population.

## Data Availability

In the interest of participant privacy and confidentiality, the data sets generated and/or analyzed in the current study are not publicly available. However, they can be obtained from the corresponding author upon reasonable request.
